# Mongolian spots as a finding in forensic examinations of possible child abuse–implications for case work

**DOI:** 10.1007/s00414-019-02208-9

**Published:** 2020-03-12

**Authors:** Mattias Kettner, Christoph G. Birngruber, Constanze Niess, Marco Baz-Bartels, Lena Bunzel, Marcel A. Verhoff, Constantin Lux, Frank Ramsthaler

**Affiliations:** 1 Institute of Legal Medicine, University Hospital Frankfurt/Main, Goethe University, Frankfurt/M, Germany; 2 Department of Child Protection/Kinderschutzambulanz, University Hospital Frankfurt/Main, Goethe University, Frankfurt/M, Germany; 3 Institute of Legal Medicine, University Hospital Giessen, Justus Liebig University, Giessen, Germany; 4 Department of Neuropediatrics, University Hospital Frankfurt/Main, Goethe University, Frankfurt/M, Germany; 5 Institute of Legal Medicine, Saarland University, Homburg, Saar Germany

**Keywords:** Mongolian spot, MS, Child abuse, Forensic examination, Nevus of Ito, Nevus of Ota

## Abstract

Mongolian spots (MS) are congenital dermal conditions resulting from neural crest-derived melanocytes migration to the skin during embryogenesis. MS incidences are highly variable in different populations. Morphologically, MS present as hyperpigmented maculae of varying size and form, ranging from round spots of 1 cm in diameter to extensive discolorations covering predominantly the lower back and buttocks. Due to their coloring, which is also dependent on the skin type, MS may mimic hematoma thus posing a challenge on the physician conducting examinations of children in cases of suspected child abuse. In the present study, MS incidences and distribution, as well as skin types, were documented in a collective of 253 children examined on the basis of suspected child abuse. From these data, a classification scheme was derived to document MS and to help identify cases with a need for recurrent examination for unambiguous interpretation of initial findings alongside the main decisive factors for re-examination such as general circumstances of the initial examination (e. g., experience of the examiner, lighting conditions) and given dermatological conditions of the patient (e. g., diaper rash).

## Introduction

Examinations of children suspected to have suffered from child abuse have been a constantly growing field of expertise in forensic routine casework with regard to frequency and extent over the past two decades. The prevalence of children aged 0–14 suffering from some kind of physical abuse has been estimated at 22.9 % in the European Union [1]. In a forensic context, examinations are usually mandated by investigative authorities or the youth welfare services. In these cases, implications of forensic expert opinion are potentially enormous including temporary or permanent removal of the child from the family home and criminal prosecution of the perpetrators. Due to the typically clandestine and domestic nature of child abuse, the number of involved persons is often limited to presumed victims of child abuse and alleged perpetrators thereof. Since many of the patients examined are infants or toddlers with limited usability of witness reports, explanatory statements concerning documented injuries are mainly constricted to alleged perpetrators and are thus also of limited value. In consideration of the aforementioned, statements claiming that a certain dermatological condition is congenital have to be scrutinized thoroughly.

Mongolian spots (MS) are a congenital dermatological condition believed to result from neural crest-derived melanocytes migrating to the skin during embryogenesis, which normally reside at the dermal-epidermal junction [2, 3]. MS incidences vary highly between different populations ranging from 0.04 to 96.53% (Table 1).

**Table 1:** Incidence of Mongolian spots in different ethnicities

	Publication year	Population	Age	n	Percentage of MS
Cordova et al. [4]	1981	US–African Am	Newborn	259	96.53
		US–Hispanic	Newborn	62	46.57
		US–Caucasian	Newborn	42	9.32
		US–Asian	Newborn	2	100
Rivers et al. [5]	1990	Australian–Caucasian	Newborn	346	13.3
		Australian–Mongolian	Newborn	56	83.9
		Australian–Australasian	Newborn	9	77.8
Karvonen et al. [6]	1992	Finnish	Newborn	4346	0.04
Tsai et al. [7]	1993	Chinese	Newborn	3345	86.3
Magana-Garcia et al. [8]	1997	Mexican	Newborn	1000	77.0
Egemen et al. [9]	2006	Turkish	1–12 months	924	26.0
Shih et al. [10]	2007	Taiwanese	Newborn	500	61.6
Fehrabas et al. [11]	2009	Turkish	Newborn	816	13.2
Reza et al. [12]	2010	Iranian	Newborn	2305	11.4
Kanada et al. [13]	2012	US–Caucasian	Newborn	263	6.7
		US–Hispanic	Newborn	116	25.7
		US–Other	Newborn	56	38
		US–Asian	Newborn	41	40.7
		US–African Am.	Newborn	19	32.1
Gupta et al. [14]	2013	Indian	Newborn	2313	65.9
Haveri et al. [15]	2014	Indian	Newborn	1000	84.7
Shehab et al. [16]	2015	Egyptian	Newborn	177	20.5
Punuru et al. [17]	2016	Indian	Newborn	100	84.0
Sandeep et al. [18]	2016	Indian	Newborn	250	61.8
Budair et al. [19]	2017	Saudi-Arabian	Newborn	313	63.07

They belong to the group of congenital dermal melanocytoses comprising MS, nevus of Ito (naevus fuscocoeruleus deltoideoacromialis), nevus of Ota (naevus fuscocaeruleus ophthalmomaxillaris), and melanocyte hamartoma [20–23]. These conditions can be distinguished primarily based on their distribution site (Table 2). Histologically, groups of spindle-shaped melanocytes percolate through the reticular dermis and subcutaneous tissue in all four entities comprised under the term dermal melanocytoses [2]. Morphologically, MS present as hyperpigmented maculae of varying size and form, ranging from round spots of 1 cm in diameter to extensive discolorations covering predominantly the lower back and buttocks in their entirety. Despite these preferential sites, the upper back [24] and scalp [25] may also be affected. They are usually colored blue-gray to blue-green but may also shade into purple, dark blue-red and brown-black tones, which is also due to differing skin types (e.g., discoloration due to MS in skin type 1 as compared to type 6 according to Fitzpatrick [26]). Superimposition of MS [27, 28] and Café-au-lait spots [29] on MS has been described. Furthermore, MS have been associated with inherited disorders such as mucopolysaccharidosis type I (Hurler syndrome) [30, 31], GM1 gangliosidosis type I [32–34], mucopolysaccharidosis [35], type II (Hunter syndrome) [36], mucolipidosis [37], Niemann-Pick disease [38], and α-mannosidosis [39]. Typically, MS regress over the first years and disappear until the age of 6 years [40]. In some cases, persistence to adult age has been reported [41].

**Table 2:** Typical distribution, age of onset, and clinical course of dermal melanocytoses

	Typical distribution	Onset	Course
Mongolian spots (MS)	Lower back and sacrococcygeal region	Congenital or early childhood	Pronounced at the age of 1 and 2, typically disappears until the age of 6 years
Nevus of Ito	Shoulder area in the distribution of the posterior supraclavicular and lateral cutaneous brachial nerves	Congenital or around puberty	Persistent
Nevus of Ota	Skin, ocular, and oral mucosal surfaces in the distribution of the ophthalmic and maxillary branches of the trigeminal nerve	Congenital or around puberty	Persistent
Melanocyte hamartoma	Dermatomal distribution	Congenital	Persistent

From a forensic perspective, MS may pose a challenge on the examiner to differentiate between possible signs of child abuse and dermatological condition [42–44]. This holds especially true since medical examinations in a forensic context are usually snapshots of a given status at a specific moment in time. Thus, MS may mimic a hematoma due to blunt force and may even be seen as proof of recurrent trauma in the presence of “additional” differently colored hematoma. In Central Europe with a population predominantly assigned to skin types I and II, incidences of MS in the overall population and thus the forensic examination sample are rather low. In late 2015, a pronounced increase in cases wit MS over the last year was noted, which was believed to be mainly due to examinations assigned by child welfare services on behalf of children in refugee families. Therefore, we conducted a prospective study to determine the frequency of MS in examinations of children with possible child abuse, to document possible challenges for the forensic examiner, and to develop a classification scheme, which may help, alongside the main decisive factors such as general circumstances of the initial examination (e. g., experience of the examiner, lighting conditions) and given dermatological conditions of the patient (e. g., diaper rash), to identify cases with a need for recurrent examination.

## Material and methods

All cases of examinations of children on the basis of possible physical or sexual abuse conducted at the Institute of Legal Medicine in Frankfurt/M were included for a time span of 2 years (October 2015–September 2017). Examinations were carried out in an examination room of the Department of child protection/Kinderschutzambulanz with constant lighting and general examination conditions over the time span of 2 years. We adopted an examination regimen with a recommended second examination conducted after a suitable time span of 2 to 6 weeks in cases displaying MS with suspected presence of both, hematoma and MS, to differentiate between these conditions. All examinations, in which Mongolian spots were seen, were documented and archived in a separate register. In addition, a search using the German translation of the words “Mongolian spot,” “Mongolian,” “dermal melanocytosis” was conducted using the database of written expert opinion statements of the Institute of Forensic Medicine to exclude possible missing entries to the register. The search was limited to statement category 128 (an internal code indicating examinations of children aged 0–18 years). Furthermore, all files of documentary photographs of cases coded 128 were screened for MS, which had not been listed in the register or described by the keywords used for the database search. For those cases not included in the register and without photographic documentation of the back region, the text files were screened for possible descriptions matching MS without stating the actual word.

In a second step, data of all cases registered under category 128 were extracted from the written forensic statements. We recorded age (in years) at the time of examination (grouped 0–3 years, 4–6 years, combined 0–6 years, and 7–18 years), population group (Caucasian, Mongolid, Negrid), skin types I–VI [26], and gender. Skin type determination was carried out by two independent observers (one physician being a member of the examination team of the institute, the other one working at another institute of legal medicine) determining skin types independently. In cases of interobserver differences, the case was assigned to a skin type by a panel of four of the authors. For descriptive and explorative statistical analysis the statistical software package MedCalc (MedCalc software bvba, Ostend, Belgium) was used. For patients with MS, written expert statements and external documents handed over to the examiners by the police or youth welfare personnel were screened for admission criteria to assess, whether MS had been mistaken for hematoma.

In a third step, cases with documented MS were examined to assess MS location(s), quantity, and dimension(s). From these data, MS constellations found in this cohort were extracted and a classification scheme was developed.

## Results

Within the 2-year period, 253 examinations of children aged 0–18 were conducted. MS were seen in 26 cases (10.27 %). In the age group 0–3 years (*n* = 98, *f* = 46, *m* = 52), 21 patients (21.4%) with MS (*f* = 7, *m* = 14) were identified, whereas in the age group 4–6 years (*n* = 40, *f* = 23, *m* = 17) 2 patients (5%, *f* = 1, *m* = 1), and in the age group 7–18 years (*n* = 115, *f* = 57, *m* = 58) 3 patients (2.6%, f = 1, m = 2, aged 7, 7, and 10 years, respectively) with MS were seen. While the arithmetic mean for all children examined in this period was 6.1 ± 4.9 years, children with MS had highly significantly differing arithmetic mean age of 2.2 ± 2.6 years (*t* = − 3.85; *p* = 0.0001).

**Fig. 1:**
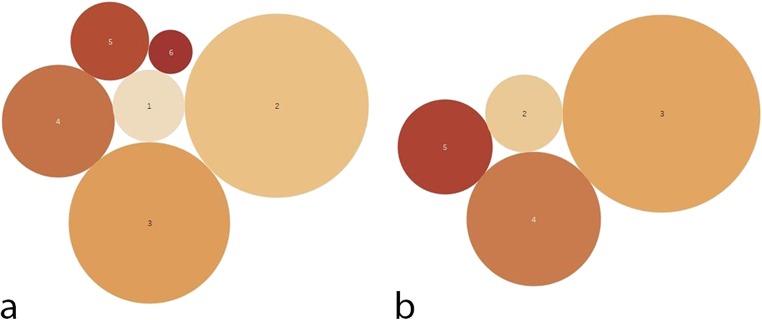
Bubble diagrams showing skin type distribution in **a** the examined collective and **b** patients with MS. Skin types are marked as numbers within bubbles

Analysis of skin type classification showed a predominance of type 2 and 3 (type 1 = 34, type 2 = 75, type 3 = 79, type 4 = 36, type 5 = 25, and type 6 = 4) (Fig. 1a) with patients displaying MS predominantly found in types 3 and 4 (type 1 = 0, type 2 = 3, type 3 = 14, type 4 = 6, type 5 = 3, and type 6 = 0) (Fig. 1b).

Comparative incidence density rate analysis revealed a grouped incidence rate for female patients of 1:14 (95 % CI 1:31–1:7) and for male patients of 1:5 (95 % CI 1:8–1:4), respectively. Incidence rates thus differed highly significantly between sexes (*p* = 0.0096, incidence rate difference − 1:9, incidence rate ratio 0.378) [45, 46]. *Χ*^2^ testing for incidence rate differences in population groups did not show a significant difference (*p* = 0.225). Comparison of incidence rates for grouped skin types (group 1 = skin types 1 + 2, group 2 = skin types 3–6) showed a highly significant difference (*p* = 0.0004, incidence rate difference − 1:7) with an incidence rate of 1:53 (95 % CI 1:438–1:15) for group 1 and 1:6 (95 % CI 1:10–1:4) for group 2.

Analysis of written expert opinions and information provided by the police and youth welfare personnel showed, that in 19 out of 26 cases, suspected hematoma had been the initial reason for admission (other primary non-exclusive admission criteria: cranial fracture/intracranial hemorrhage = 2, limb fracture = 3, thermal injuries = 3). In 2 cases only MS were found, in only 1 case MS had been described as such clinically. In 8 cases, patients had a follow-up examination after 2–6 weeks to clarify the status of hematoma-suspicious findings. In 2 cases, photographs of examinations, that had taken place 3 days and 1 week in advance, were sent in for evaluation after the physical examination.

**Fig. 2:**
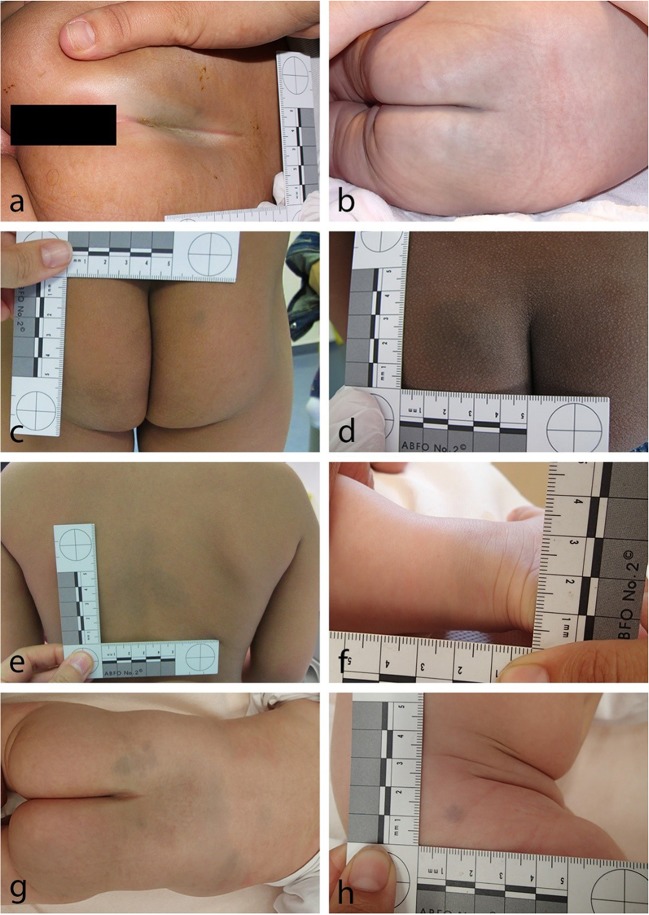
Classification of MS: Type Ia MS with common distribution at the tip of the intergluteal cleft (**a** small, **b** large). **a** Small type Ib MS of the right buttock in the presence of a hematoma of the **c** left buttock and **d** large type Ib MS of the left buttock. **e** Relatively large type IIa MS of the upper back and **f** rather small type IIb_left ankle MS of the lower limb. Type III (Ib/IIab_right thigh) MS in a child with three follow-up examinations

**Fig. 3:**
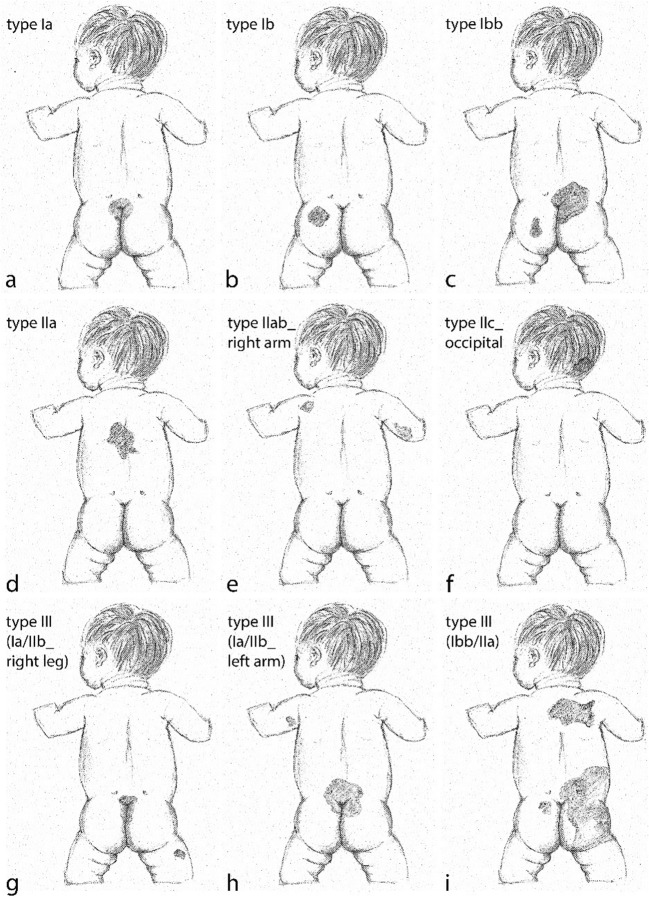
Classification of MS: type I MS localized in the buttocks region (**a** type Ia with symmetrical MS at the tip of the intergluteal cleft; **b** type Ib with asymmetrical MS of the left buttock; **c** type Ibb with two asymmetrical MS). **d** Type IIa MS located at the upper back, **e** type IIab with MS located at the upper back and right arm, and **f** type IIc with a MS located occipitally; **g**–**i** type III MS displaying combinations of type I and type II MS

The inspection of photographic case documentations showed different but distinguishable MS patterns with most cases (in our study) displaying a round to oval sometimes spindle-shaped MS mostly symmetrically arranged at the tip of the intergluteal cleft varying in size between 1 cm and about 6 cm in diameter (Fig. 2a,b). Further, MS were seen in varying sizes covering parts of the buttocks (Fig. 2c,d). In one case, the complete buttocks showed blueish discolorations. In a number of cases, MS were seen on parts of the back or as a combination on the buttocks, the back as well as the limbs (Fig. 2e–h). In consideration of these findings, a talking alphanumerically coded classification scheme was developed (Fig. 3), which was intended to encompass all possible MS constellation seen in our study and reported in the literature, to enable a clear risk stratification for the forensic expert, and to produce a speaking classification type, which documents a status, that may later be compared to a given status upon a follow-up examination of a child. Type I consists of MS found on the buttocks, with subtype a found at the tip of the intergluteal cleft, displaying a symmetrical median/paramedian distribution. Subtype b shows MS of varying sizes on the buttocks in an asymmetrical distribution. Existence of more than one subtype or more than one MS of a specific subtype is coded in the alphabetical part of the code, e.g., Type I bb for one MS asymmetrically located at the intergluteal cleft and an additional MS at other parts of the buttocks. Type II shows MS found on other parts of the body, mainly the upper back (subtype a). In our sample, we found one case with MS on a lower limb, which we classified as subtype b (location at lower or upper limbs). In the literature, there has been a report on MS seen on the scalp [25], which may then be subsumed under subtype c (location other than subtype a and b). Again, multiple MS in a specific region are documented as in type I. Type III is a combination of MS found in two regions, the buttocks and any other part of the body. It may best be coded as, e.g., type III (Ia/II left elbow).

## Discussion

In the present prospective study, examining MS in a collective of 253 children examined due to suspected physical child abuse, MS of varying sizes and shapes were found in 10.27% (21.4% age group 0–3 years) of the cases. This incidence density rate is comparable to those found in Caucasian collectives in earlier studies [5, 13]. The examined patients comprised Caucasian, negrid, and mongolid ancestry, and thus reflect a typical central European patient spectrum seen in forensic examinations. Arithmetic mean age for all patients of this cohort was just above 6 years, while mean age of patients displaying MS was just above 2 years. This finding is in good accordance to observations, that congenital MS usually disappear until the age of 6 years [25] and are seldomly observed afterward [41]. In this study, a clear preponderance of MS seen in examinations of male patients and patients of grouped skin types 3–6 of the Fitzpatrick classification as compared to grouped skin types 1 and 2 was noted, which has been reported before on the basis of ancestral grouping.

MS are a congenital condition that is well known in the clinical setting. Despite this, MS as a clinical finding in a forensic setting was described in only one case, which may either be due to neglecting a finding seen as non-relevant or overseeing it. In 2 of the 26 MS cases, MS were the only finding seen during the physical examination ordered on the basis of suspected hematoma, while in 18 cases MS had not been differentiated from hematoma clinically. In 8 of the MS cases, a follow-up examination was assigned, leading to unambiguous assessment of MS as compared to hematoma. This shows that MS may be misinterpreted and that attention of forensic experts needs to be drawn to MS and to follow-up examinations, helping to avoid possible double-misinterpretations (clinically and forensically) with potentially dramatic unjustified consequences for affected families.

On the basis of our findings, we developed a talking alphanumerical classification system of MS intended to enable the clinical and forensic examiner to document a given status for subsequent comparison. As MS are known to be found predominantly on the buttocks and lower back, type 1 resembles this most frequent distribution type with subtype a being centrally located at the tip of the intergluteal cleft comprising various sizes, and subtype b being located on the buttocks or lower back asymmetrically and in various sizes. In order to document the correct numbers, the alphabetical code is repeated for additional MS (e.g., type Iabb). Type II comprises all MS seen on body parts other than the buttocks and lower back, i.e., mainly on the upper back (subtype a) or the remaining body locations (subtype b). In this respect, we had to choose between a clear and decisive classification leaving some anatomical data to be added and a classification with a very detailed, yet confusing anatomical code. For practical purposes, we suggest to add anatomical information on the specific location in these rare cases (e.g., type IIb_left ankle). Type III had to be included to clearly mark a combination of types I and II, which may be documented as type III (Ia/IIb_left ankle) for a patient showing MS at the tip of the intergluteal cleft as well as the left ankle. A coded description of MS area was discussed but not deemed appropriate, since MS fade out over time and may thus be seen covering less skin area at an examination conducted after a relevant time span (of e.g., 2 years). At least in our collective, it was possible to differentiate between inconspicuous findings (type Ia) and those, in which a follow-up examination seemed advisable in the absence of further information indicating MS persistence at a specific location (types Ib, II, III), e.g., hospital reports or photographic documentations. Nevertheless, a decision, whether a second examination seems advisable and should be exerted, cannot be solely based on a classification scheme but has to include the main decisive factors such as the experience of the examiner, general examination conditions of the first examination such as the lighting, as well as given dermatological conditions of the patient in the respective areas, e.g., a diaper rash.
